# Supplementary material for: Tsunamites versus tempestites: Various types of redeposited stromatoporoid beds in the Devonian of the Holy Cross Mountains (Poland), a case study from the Ołowianka Quarry
Source: PLoS One. 2022 May 17;17(5):e0268349. doi: 10.1371/journal.pone.0268349 (PMC9113602; doi:10.1371/journal.pone.0268349)
Supplement: Attachment — Submitted filename: Response to Reviewers.docx [file pone.0268349.s001.docx]

**Response to Reviewers**

(Rebuttal letter)

**I. Points raised by the academic editor**

**1.** Please ensure that your manuscript meets PLOS ONE's style requirements, including those for file naming.

The manuscript has been checked, and all style requirements, including file naming, are being met.

**2.** In your Methods section, please provide additional information regarding the permits you obtained from the landowner for carrying out the work on this land ….. if no permits were required, a brief statement explaining why.

The following statement was added in the “Materials and methods” section: Fieldwork was conducted in the abandoned Ołowianka Quarry. Exploitation in the quarry ceased in the 1980s: today, it is state owned, barren land with free access to the general public. According to Polish law, no permits are required to perform geological studies in such an area

**3.** We note that the grant information you provided in the ‘Funding Information’ and ‘Financial Disclosure’ sections do not match. When you resubmit, please ensure that you provide the correct grant numbers for the awards you received for your study in the ‘Funding Information’ section.

Corrected. Now the Funding statement should read as follows:

The study was financed from the funds of the Faculty of Geology, University of Warsaw. (geo.uw.edu.pl) Grant number 501-0113-01-1130203

The funders had no role in study design, data collection and analysis, decision to publish, or preparation of the manuscript

**4.** Please remove any funding-related text from the manuscript and let us know how you would like to update your Funding Statement.

The funding-related text has been removed from the manuscript.

**5.** We noted in your submission details that a portion of your manuscript may have been presented or published elsewhere. ( The manuscript has been presented to dr Mikołaj Zapalski, PLOS One editor, who’s preliminary opinion was that it is suitable for submission in your Journal.) Please clarify whether this publication was peer-reviewed and formally published. If this work was previously peer-reviewed and published, in the cover letter please provide the reason that this work does not constitute dual publication and should be included in the current manuscript.

The work has not been peer reviewed or published in any way. The mentioning of presenting the paper to dr Mikołaj Zapalski, PLOS One editor, meant only that I asked his personal advice, based on his experience as a PLOS editor, if the topic and volume of the paper generally fits the expectations and requirements of your journal. He has just glanced the text, did not check it, or make any corrections.

**6.** We note that you have stated that you will provide repository information for your data at acceptance. Should your manuscript be accepted for publication, we will hold it until you provide the relevant accession numbers or DOIs necessary to access your data. If you wish to make changes to your Data Availability statement, please describe these changes in your cover letter and we will update your Data Availability statement to reflect the information you provide.

The only data I have collected, which eventually can be examined by other workers, other than field photographs presented in the figures, are the thin plates illustrated on figure 7. All other discussed data are observations made in field.

Therefore, please put the following information in the Data Availability Statement “The studied thin plates illustrated on figure 7 are deposited and available in the S.J. Thugutt Geological Museum, Faculty of Geology, University of Warsaw, Poland (MWGUW) under the inventory number: MWGUW 009779.

7. We note that Figures 1, 2, and 3 in your submission contain map images, which may be copyrighted. Please upload the completed Content Permission Form or other proof of granted permissions as an "Other" file with your submission. In the figure caption of the copyrighted figure, please include the following text: “Reprinted from [ref] under a CC BY license, with permission from [name of publisher], original copyright [original copyright year].”

Copyright permissions have been obtained from the copyright holders (files “Other”) . However, figures 1, 2, and 3 have been changed, according to the suggestions of one of the reviewers (figures 1 and 2 have been merged into one). None of these figures simply copy the figures from previous papers (including maps), but are constructed by myself based on previous papers, with considerable changes. Therefore, in the captions I did not write “reprinted from…..” but “modified after…..”

8. Please review your reference list to ensure that it is complete and correct.

The reference list has been thoroughly checked

**II. Points raised by reviewer #1**

1. Polishing the language and correct some grammar mistakes

The whole text has been corrected by a native speaker

2. Some paragraphs, especially in the morphometrical analysis and results, can be reduced or combined, so that the entire manuscript is more clear in structure.

The structure has been left as previously

3. Thin section photos are not quite satisfactory and can be improved by adjusting contrasts and brightness.

Thin section photos have been improved (figure 6, according to new numeration)

4. The field photos can be replaced with colors.

Done

5. Some figures, such as figure 1 and 2, and figure 3 and 6 can be combined or adjusted.

Figures 1 and 2 have been merged into one figure. Figures 3 and 6 ( 2 and 5 according to new numeration) remain separate

**III. Points raised by reviewer #2**

**1.** Some English expressions are too complicated …. concise language is required.

The whole text has been corrected by a native speaker. All the specific points, with reference to particular lines (647-648, 83-85, 393-396, 428-431, 541, 823-825, and 887) have been corrected.

2. The demonstration on the relationship between shape and growth form of stromatoporoid is unclear.

I hope now it is clear. The topic has been discussed in a number of papers cited in the text

3. The conclusion is disorganization.

I don’t understand this comment

4. Line 341-342. Relative explains of Figure 7 G, H are missing. The sentence of “The wall on A is approximately 4m high, the visible part of the wall on D is approximately 3m high” is very confused.

Figures 7 and 8 have been switched. Now the captions comply with the figures

5. Figure 7. The explanations of scale bars are missing.

Figures 7 and 8 have been switched. Scale bars on new figure 6 have been explained

6. The explanation of “----------” in Table 2 is missing.

This simply means that this particular feature does not apply here. Some features can be interpreted in terms of stromatoporoid growth environment, some in terms of transport and final accumulation, but not all in terms of both these aspects. I don’t feel this needs explanation in the table captions.
